# Socioeconomic Status and Health Communication Inequalities in Japan: A Nationwide Cross-Sectional Survey

**DOI:** 10.1371/journal.pone.0040664

**Published:** 2012-07-12

**Authors:** Yoshiki Ishikawa, Hiromu Nishiuchi, Hana Hayashi, Kasisomayajula Viswanath

**Affiliations:** 1 Department of Public Health, Jichi Medical University, Shimotuke-shi, Tochigi, Japan; 2 Department of Preventive Medicine and Public Health, School of Medicine, Keio University, Shinjuku-ku, Tokyo, Japan; 3 Department of Society, Human Development and Health, Harvard School of Public Health, Boston, Massachusetts, United States of America; 4 Public Health Department, McCann Healthcare Worldwide Japan, Inc, Minato-ku, Tokyo, Japan; 5 Center for Community-Based Research, Dana-Farber Cancer Institute, Boston, Massachusetts, United States of America; Tehran University of Medical Sciences, Iran (Islamic Republic of)

## Abstract

**Background:**

Considerable evidence suggests that communication inequality is one potential mechanism linking social determinants, particularly socioeconomic status, and health inequalities. This study aimed to examine how dimensions of health communication outcomes (health information seeking, self-efficacy, exposure, and trust) are patterned by socioeconomic status in Japan.

**Methods:**

Data of a nationally representative cross-sectional survey of 2,455 people aged 15–75 years in Japan were used for secondary analysis. Measures included socio-demographic characteristics, subjective health, recent health information seeking, self-efficacy in seeking health information, and exposure to and trust in health information from different media.

**Results:**

A total of 1,311 participants completed the questionnaire, giving a response rate of 53.6%. Multivariate logistic regression revealed that education and household income, but not employment, were significantly associated with health information seeking and self-efficacy. Socioeconomic status was not associated with exposure to and trust in health information from mass media, but was significantly associated with health information from healthcare providers and the Internet.

**Conclusion:**

Health communication outcomes were patterned by socioeconomic status in Japan thus demonstrating the prevalence of health communication inequalities. Providing customized exposure to and enhancing the quality of health information by considering social determinants may contribute to addressing social disparities in health in Japan.

## Introduction

There is mounting evidence of influence of socioeconomic status (SES) on health, making health inequality one of the major public health problems worldwide [1]. Increases in social and health inequalities have been reported in Japan and other developed countries recently [2], [3]. The Gini Coefficient, a measure of inequality in income distribution, has been consistently increasing from 0.312 in 1995 to 0.454 in 2008, indicating the widening of the income inequality gap in Japan [4]. While studies have attempted to delineate the influence of socioeconomic differences on mortality, morbidity, or risk factors, Japan may not necessarily reflect the same pattern of relationships as other developed countries [2]. For example, an association between higher education attainment and health is not strongly expressed among the Japanese [2], [5]. A greater understanding of the unique mechanism linking social determinants to health is necessary to reduce the widening health inequality in Japan.

The Structural Influence Model (SIM) has proposed communication inequality to be one of the mechanisms linking SES and health inequalities [6]. According to this model, differences in health and preventive behaviors among different social groups may be partly explained by focusing on how social determinants of health, such as income, education, and employment are related to health communication outcomes – how people access, seek, process, and act on heath information [7], [8]. Previous research has suggested that SES is related to exposure and attention to, trust in, and use of health information, which is in turn related to health-related behaviors such as fruit and vegetable consumption, physical activity, sun protection, and smoking [9]–[15]. Therefore, an understanding of disparities in health communication outcomes may contribute to the development of communication strategies that could address health inequalities worldwide.

As limited research on health communication inequalities has been conducted outside the United States, the purpose of this study is to use nationwide cross-sectional data from Japan to examine the relationship between SES and health communication outcomes, including health information seeking, and exposure to and trust in health information from various media. It is hoped that findings from our study will (a) better elucidate how health communication inequalities may link social determinants and health, (b) as a more readily addressable social determinant, may provide direction for intervention to address inequalities in Japan.

## Materials and Methods

### Data Source

Data of the 2009 National Healthy Lifestyle Survey (NHLS) by the Ministry of Health, Labour and Welfare (Ministry of Health, Labour and Welfare, 2010) were used for analysis in the present study. The NHLS is a nation-wide cross-sectional survey conducted in August 2009. A two-stage stratified random sampling method was used to select 2455 people aged from 15–75 years in Japan. Household drop-off surveys were then conducted with the selected sample. Questionnaires were hand-delivered by trained research staff and collection occurred later. In total, 1311 people completed the questionnaire, corresponding to a response rate of 53.6%. Respondents were anonymized during data analysis.

### Measures

Socio-demographic characteristics, subjective health status and health communication outcomes were measured. Socio-demographic variables included sex, age, education, household income, employment status, and marital status. Information on nationality or migration was not collected as Japan is considered as a relatively homogeneous society, with the migrant population making up only one percent of the overall population.

Age was categorized as 15–19, 20–29, 30–39, 40–49, 50–59, 60–69, or 70–75 years old. Education was assessed by the question “What is the highest level of education that you have completed?” followed by the following response options: (1) high school or less; (2) some college or technical school; (3) 4-year college degree or more. Yearly household income was categorized as (1) less than 3.5 million yen (around 28 000 USD); (2) 3.5 to 7.5 million yen (around 28 000 USD to 60 000 USD); (3) more than 7.5 million yen (more than 60 000 USD). Employment status was categorized as (1) regular employment; (2) irregular or part-time employment; (3) self-employed; (4) unemployed/others. Subjective health status was assessed by the question “Please rate your feelings of health on a five-point scale from good to poor.”, and was categorized as (1) good; (2) fair; (3) poor.

Four dimensions of health communication outcomes were assessed: (1) exposure to health information sources; (2) health information seeking; (3) self-efficacy in seeking health information, and (4) trust in health information sources. Exposure to health information sources was assessed as a dichotomous (yes/no) variable with the statement “Have you received information useful to health from the following sources in the past 6 months?” Health information seeking was assessed as a dichotomous (yes/no) variable with the statement “Have you looked for information about health or medicine recently?” Self-efficacy in seeking health information was assessed by the question “Do you have confidence in getting information or advice about health when you need it?” followed by five response options: ‘very confident’, ‘somewhat confident’, ‘don’t know’, ‘somewhat not confident’, or ‘not confident’. Trust in health information was assessed as a dichotomous (yes/no) variable with the statement “Do you trust information about health or medicine from the following sources?” Health information sources included friends and relatives, radio, TV news, TV information shows, newspapers, magazines, books, health care provider, Internet websites, and community newsletters. Participants who have not received any information about health or medicine in the past 6 months were asked to select ‘none’ in response to this question.

Missing values in education, household income, employment status, marital status, subjective health status and self-efficacy in health information seeking were imputed by “some college”, “$28,000 to < $60,000”, “Unemployed/other”, “Not married”, “Fair” and “Don’t know” respectively as the single imputation. Also, missing values in recent seeking of health information, exposure to and trust in health information sources were imputed by “no”.

### Ethics

Based on ethical guidelines in Japan, ethical review was not undertaken for this study with the following reasons: 1) this study was a secondary analysis of publicly available data obtained as part of governmental surveillance; 2) the authors are researchers independent from the government agencies that conducted the survey; and 3) the authors report no conflict of interests related to this study.

### Statistical Analysis

Multivariate logistic regression was conducted to examine differences in health communication outcomes by socio-demographic factors. Education, household income and employment status were used as independent variables and the models were adjusted for sex, age, marital status, and subjective health status. SAS 9.1.3 (SAS Institute, Cary, NC) was used for all statistical analyses.

## Results

### Descriptive Data

Participant characteristics are reported in Table 1. Approximately half of the participants have high school education or less, 24.0% have some college education, and 23.5% have a college degree. Almost one-quarter of participants earned household income of less than 28 000 USD (3.5 million JPY) per year, 56.4% earned 28 000–60 000 USD (3.5–7.5 million JPY) per year, and 17.4% earned more than 60 000 USD (7.5 million JPY) per year. Close to forty percent of the respondents replied they feel ‘good’ about their health.

**Table 1:** Frequencies and percentages of demographic variables.

		Sample Population		Japanese Population
Variable	Item	n	(%)	(%)
Total		1311	(100.0)	
Sex	Male	654	(49.9)	(49.8)^a^
	Female	657	(50.1)	(50.2)^a^
Age	15–19	82	(6.3)	(6.3)^a^
	20–29	198	(15.1)	(14.0)^a^
	30–39	250	(19.1)	(18.7)^a^
	40–49	219	(16.7)	(17.4)^a^
	50–59	233	(17.8)	(17.1)^a^
	60–69	256	(19.5)	(19.2)^a^
	70–75	73	(5.6)	(7.3)^a^
Education	High school or less	689	(52.6)	(61.6)^a^
	Some college	314	(24.0)	(16.4)^a^
	College graduate	308	(23.5)	(22.9)^a^
Household income	≧?$60 000	228	(17.4)	(23.6)^b^
	$28 000 to < $60 000	740	(56.4)	(37.7)^b^
	< $28 000	343	(26.2)	(38.7)^b^
Employment status	Regular	419	(32.0)	(32.1)^a^
	Irregular/part-time	338	(25.8)	(16.4)^a^
	Self-employed	175	(13.3)	(8.5)^a^
	Unemployed/other	379	(28.9)	(43.1)^a^
Marital status	Married	915	(69.8)	(59.5)^a^
Subjective health	Good	522	(39.8)	(36.8)^b^
	Fair	605	(46.1)	(50.3)^b^
	Poor	184	(14.0)	(12.9)^b^

a Population Census, 2010.

b Comprehensive Survey of Living Conditions, 2010.

A comparison of our sample group with the census data revealed comparable distribution in terms of age, sex, regular employment, and subjective health status. However, our sample included more people from the middle class in terms of education and household income.

### Exposure to Health Information Sources

Respondents were exposed to health information mainly via TV information shows (n = 670, 51.1%), friends and relatives (n = 446, 34.0%), and newspapers (n = 412, 31.4%) (Table 2).

**Table 2:** Frequencies and percentages of health communication outcomes.

				Sex					Age						
				Male		Female		p	15–39		40–59		60–75		p
		n	(%)	n	(%)	n	(%)		n	(%)	n	(%)	n	(%)	
Total		1311	(100.0)	654	(100.0)	657	(100.0)		530	(100.0)	452	(100.0)	329	(100.0)	
Recent seeking of	Yes	342	(26.1)	159	(24.3)	183	(27.9)	0.153	120	(22.6)	132	(29.2)	90	(27.4)	0.051
health information															
Health information seeking	Very/Somewhat	661	(50.4)	349	(53.4)	312	(47.5)	0.101	274	(51.7)	246	(54.4)	141	(42.9)	0.027
self-efficacy	Don’t know	511	(39.0)	241	(36.9)	270	(41.1)		201	(37.9)	164	(36.3)	146	(44.4)	
	Somewhat not/Not	139	(10.6)	64	(9.8)	75	(11.4)		55	(10.4)	42	(9.3)	42	(12.8)	
Exposure (TV information shows)	Yes	670	(51.1)	312	(47.7)	358	(54.5)	0.014	256	(48.3)	242	(53.5)	172	(52.3)	0.232
Exposure (friends and relatives)	Yes	446	(34.0)	181	(27.7)	265	(40.3)	<.0001	180	(34.0)	166	(36.7)	100	(30.4)	0.183
Exposure (newspapers)	Yes	412	(31.4)	203	(31.0)	209	(31.8)	0.764	105	(19.8)	168	(37.2)	139	(42.2)	<.0001
Exposure (TV news)	Yes	357	(27.2)	184	(28.1)	173	(26.3)	0.464	168	(31.7)	107	(23.7)	82	(24.9)	0.011
Exposure (internet website)	Yes	323	(24.6)	175	(26.8)	148	(22.5)	0.075	164	(30.9)	129	(28.5)	30	(9.1)	<.0001
Exposure (magazines)	Yes	299	(22.8)	124	(19.0)	175	(26.6)	0.001	136	(25.7)	109	(24.1)	54	(16.4)	0.005
Exposure (healthcare provider)	Yes	250	(19.1)	133	(20.3)	117	(17.8)	0.244	60	(11.3)	89	(19.7)	101	(30.7)	<.0001
Exposure (community newsletters)	Yes	239	(18.2)	94	(14.4)	145	(22.1)	0.000	51	(9.6)	91	(20.1)	97	(29.5)	<.0001
Exposure (books)	Yes	175	(13.3)	84	(12.8)	91	(13.9)	0.592	72	(13.6)	63	(13.9)	40	(12.2)	0.754
Exposure (none)	Yes	145	(11.1)	89	(13.6)	56	(8.5)	0.003	74	(14.0)	37	(8.2)	34	(10.3)	0.014
Exposure (radio)	Yes	123	(9.4)	72	(11.0)	51	(7.8)	0.044	28	(5.3)	63	(13.9)	32	(9.7)	<.0001
Trust (healthcare provider)	Yes	1185	(90.4)	579	(88.5)	606	(92.2)	0.023	486	(91.7)	412	(91.2)	287	(87.2)	0.077
Trust (community newsletters)	Yes	948	(72.3)	444	(67.9)	504	(76.7)	0.000	374	(70.6)	348	(77.0)	226	(68.7)	0.019
Trust (TV news)	Yes	890	(67.9)	433	(66.2)	457	(69.6)	0.194	365	(68.9)	306	(67.7)	219	(66.6)	0.777
Trust (TV information shows)	Yes	840	(64.1)	394	(60.2)	446	(67.9)	0.004	350	(66.0)	279	(61.7)	211	(64.1)	0.373
Trust (friends and relatives)	Yes	818	(62.4)	378	(57.8)	440	(67.0)	0.001	346	(65.3)	277	(61.3)	195	(59.3)	0.175
Trust (book)	Yes	816	(62.2)	396	(60.6)	420	(63.9)	0.207	346	(65.3)	290	(64.2)	180	(54.7)	0.005
Trust (newspaper)	Yes	799	(60.9)	383	(58.6)	416	(63.3)	0.078	336	(63.4)	267	(59.1)	196	(59.6)	0.322
Trust (radio)	Yes	710	(54.2)	346	(52.9)	364	(55.4)	0.364	275	(51.9)	252	(55.8)	183	(55.6)	0.397
Trust (magazines)	Yes	605	(46.1)	286	(43.7)	319	(48.6)	0.080	274	(51.7)	201	(44.5)	130	(39.5)	0.002
Trust (internet website)	Yes	528	(40.3)	270	(41.3)	258	(39.3)	0.457	235	(44.3)	199	(44.0)	94	(28.6)	<.0001

Table 2 shows that female are more likely to receive health information from TV information shows (p = 0.014), friends and relatives (p<0.001), magazines (p = 0,001), and community newsletters (p = 0.000), while male are more likely to receive such information from radio (p = 0.044). Also, there are differences among different age group in terms of exposure to health information; younger people are more likely to receive health information from TV news, internet website and magazines; the middle-aged respondents from radio; whereas elderly from newspapers, healthcare provider and community newsletters.

Table 3 indicates that lower education is associated with lower exposure to health information sources. Participants with high school education or less have significantly less exposure to health information from friends and relatives (OR = 0.73, 95% CI: 0.53–0.99), TV information shows (OR = 0.74, 95% CI: 0.55–0.99), healthcare providers (OR = 0.46, 95% CI: 0.32–0.67) and Internet websites (OR = 0.37, 95% CI: 0.27–0.52), compared with participants who have higher levels of education. In addition, participants with lower levels of education are more likely than college graduates to report receiving no health information through any channels (some college OR = 1.91, 95% CI: 1.04–3.51; high school or less OR = 2.24, 95% CI: 1.33–3.78). Compared with the highest income group, the group with lower household income is associated with less exposure to health information sources such as magazines, healthcare providers, and Internet websites (Table 3). Participants with no employment have significantly less exposure to health information from Internet websites (OR = 0.64; 95% CI: 0.43–0.95) than those who are employed.

**Table 3:** Logistic regression of exposure to health information sources and socio-demographic status.

	Exposure (friends and relatives)			Exposure (radio)			Exposure (TV news)			Exposure (TV information shows)		
	OR	CI	p	OR	CI	p	OR	CI	p	OR	CI	p
Education												
College graduate	1 (Reference)			1 (Reference)			1 (Reference)			1 (Reference)		
Some college	0.80	0.56–1.15	0.227	1.20	0.64–2.27	0.574	1.10	0.76–1.59	0.626	0.87	0.62–1.23	0.436
High school or less	0.73	0.53–0.99	0.044	1.64	0.96–2.80	0.072	0.94	0.68–1.31	0.728	0.74	0.55–0.99	0.044
Household income												
<$>\scale 80%\raster="rg1"<$> $60 000	1 (Reference)			1 (Reference)			1 (Reference)			1 (Reference)		
$28 000 to < $60 000	1.08	0.78–1.51	0.640	1.49	0.83–2.69	0.181	0.98	0.70–1.39	0.918	0.88	0.64–1.20	0.411
< $28 000	1.20	0.81–1.78	0.363	1.59	0.80–3.18	0.190	1.09	0.72–1.64	0.690	0.85	0.59–1.24	0.403
Employment status												
Regular	1 (Reference)			1 (Reference)			1 (Reference)			1 (Reference)		
Irregular/part-time	0.89	0.63–1.26	0.521	1.23	0.68–2.22	0.495	0.82	0.57–1.19	0.295	0.99	0.71–1.38	0.958
Self-employed	1.05	0.71–1.56	0.815	1.38	0.77–2.47	0.277	0.86	0.57–1.31	0.487	1.06	0.74–1.54	0.741
Unemployed	0.77	0.54–1.09	0.134	1.14	0.63–2.07	0.666	0.99	0.69–1.41	0.939	1.09	0.79–1.51	0.613

	Exposure (newspapers)			Exposure (magazines)			Exposure (books)			Exposure (healthcare privider)			
	OR	CI	p	OR	CI	p	OR	CI	p	OR	CI		p
Education													
College graduate	1 (Reference)			1 (Reference)			1 (Reference)			1 (Reference)			
Some college	0.88	0.60–1.29	0.526	1.21	0.82–1.79	0.334	1.33	0.83–2.12	0.232	0.62		0.40–0.95	0.029
High school or less	1.03	0.74–1.42	0.881	0.83	0.58–1.18	0.297	0.73	0.47–1.13	0.158	0.46		0.32–0.67	<0.001
Household income													
<$>\scale 80%\raster="rg1"<$> $60 000	1 (Reference)			1 (Reference)			1 (Reference)			1 (Reference)			
$28 000 to < $60 000	0.89	0.64–1.25	0.512	0.66	0.47–0.94	0.021	1.39	0.86–2.25	0.176	0.67		0.46–0.98	0.039
< $28 000	0.79	0.52–1.19	0.260	0.63	0.41–0.97	0.036	1.50	0.85–2.63	0.161	0.59		0.36–0.95	0.029
Employment status													
Regular	1 (Reference)			1 (Reference)			1 (Reference)			1 (Reference)			
Irregular/part-time	0.98	0.68–1.42	0.930	0.96	0.64–1.42	0.822	0.76	0.47–1.24	0.273	0.69		0.44–1.08	0.105
Self-employed	0.83	0.55–1.25	0.366	1.02	0.65–1.61	0.929	0.94	0.55–1.62	0.835	0.92		0.57–1.47	0.713
Unemployed	1.14	0.79–1.64	0.499	1.21	0.82–1.78	0.331	1.13	0.71–1.78	0.610	0.84		0.55–1.29	0.431

	Exposure (Internet website)			Exposure (community newsletters)			Exposure (none)		
	OR	CI	p	OR	CI	p	OR	CI	p
Education									
College graduate	1 (Reference)			1 (Reference)			1 (Reference)		
Some college	0.68	0.47–0.99	0.043	0.88	0.55–1.43	0.610	1.91	1.04–3.51	0.036
High school or less	0.37	0.27–0.52	<0.001	1.17	0.78–1.77	0.453	2.24	1.33–3.78	0.002
Household income									
<$>\scale 70%\raster="rg1"<$> $60 000	1 (Reference)			1 (Reference)			1 (Reference)		
$28 000 to < $60 000	0.52	0.37–0.72	<0.001	0.94	0.62–1.42	0.762	1.30	0.74–2.28	0.363
< $28 000	0.56	0.36–0.86	0.008	0.70	0.42–1.16	0.169	1.33	0.71–2.50	0.375
Employment status									
Regular	1 (Reference)			1 (Reference)			1 (Reference)		
Irregular/part-time	0.78	0.53–1.15	0.201	1.25	0.79–1.97	0.336	1.28	0.76–2.14	0.354
Self-employed	0.66	0.42–1.04	0.072	0.85	0.50–1.46	0.556	1.27	0.72–2.24	0.419
Unemployed	0.64	0.43–0.95	0.026	1.45	0.92–2.29	0.108	0.99	0.59–1.67	0.974

Note: All models are additionally adjusted for gender, age, marital status, and subjective health status.

### Health Information Seeking

Approximately one-fourth of participants reported having sought health information recently (Table 2). There was no difference in recent seeking of health information between male and female (p = 0.153) and among different age groups (p = 0.051).

Table 4 shows that lower levels of education and household income were associated with less recent seeking of health information. The odds of people with high school education or less having recently sought health information were 0.54 times lower than that of those who have a college degree (95% CI: 0.38–0.75). With regards to household income, a decreased likelihood of having recently sought health information seeking was found among participants in the middle income group when compared with the high income group (OR = 0.69, 95% CI: 0.49–0.97). Employment status had no significant association with health information seeking behavior.

**Table 4:** Logistic regression of health information seeking and socio-demographic status.

			Recent health information seeking									Health information seeking self-efficacy									
			OR			CI				p		OR				CI				p	
Education																					
College graduate			1 (Reference)									1 (Reference)									
Some college			0.96	0.66		–	1.39			0.834		0.80		0.57		–	1.13			0.208	
High school or less			0.54	0.38		–	0.75			<0.001		0.48		0.36		–	0.65			<0.001	
Household income																					
<$>\scale 80%\raster="rg1"<$> $60 000			1 (Reference)									1 (Reference)									
$28 000 to < $60 000		0.69			0.49			–	0.97		0.034		0.67		0.49			–	0.91		0.011
< $28 000			0.87	0.58		–	1.32			0.524		0.58		0.40		–	0.83			0.003	
Employment status																					
Regular			1 (Reference)									1 (Reference)									
Irregular/part-time			0.69	0.47		–	1.01			0.054		0.81		0.59		–	1.11			0.188	
Self-employed			0.81	0.53		–	1.24			0.334		1.08		0.75		–	1.56			0.674	
Unemployed/other			0.75	0.51		–	1.09			0.130		1.00		0.73		–	1.37			0.979	

Note: All models are additionally adjusted for gender, age, marital status, and subjective health status.

### Self-efficacy in Seeking Health Information

Nearly half of the participants had self-efficacy in seeking health information (Table 2). Although there was no difference in health information seeking self-efficacy between male and female (p = 0.101), differences were found among different age groups (p = 0.027): the elderly were less likely to have a confidence in seeking health information.

Table 4 shows that lower levels of education and household income were associated with lower self-efficacy in seeking health information. Participants with high school education or less had significantly less self-efficacy in seeking health information (OR = 0.48, 95% CI: 0.36–0.65) when compared with respondents who have a college degree. Also, respondents with mid- or low income had less self-efficacy in seeking health information (OR = 0.67, 95% CI: 0.49–0.91; OR = 0.58, 95% CI: 0.40–0.83 respectively). Employment status had no significant association with self-efficacy in seeking health information.

### Trust in Health Information Sources

Participants have a high level of trust in health information from healthcare providers (n = 1185, 90.4%), community newsletters issued by their local government (n = 948, 72.3%), and TV news (n = 890, 67.9%) (Table 2).

Female exhibit higher trust in health information from healthcare providers (p = 0.023), community newsletters (p = 0.000), TV information shows (p = 0.004), and friends and relatives (p = 0.001) when compared with male (Table 2). There are also differences between age groups in terms of trust in health information – the elderly respondents are less likely to trust books, magazines, and Internet websites when compared with young and middle-aged participants.

Participants with high school education or less reported less trust in Internet websites (OR = 0.68, 95% CI: 0.51–0.92) (Table 5) than those with higher education. Lower household income is associated with less trust in a variety of health information sources, including newspapers, magazines, books, and healthcare providers. Self-employed participants reported less trust in Internet websites (OR = 0.65, 95% CI: 0.44–0.95) and community newsletters published by local governments (OR = 0.65, 95% CI: 0.44–0.97).

**Table 5:** Logistic regression of trust in health information sources and socio-demographic status.

	Trust (friends and relatives)				Trust (radio)				Trust (TV news)				Trust (TV information shows)			
	OR		CI	p	OR		CI	p	OR		CI	p	OR		CI	p
Education																
College graduate	1 (Reference)				1 (Reference)				1 (Reference)				1 (Reference)			
Some college	1.30	0.91–1.84		0.150	1.12	0.80–1.57		0.516	1.06	0.73–1.53		0.768	1.08	0.76–1.54		0.657
High school or less	1.02	0.76–1.38		0.877	1.03	0.77–1.38		0.843	0.92	0.67–1.25		0.580	1.05	0.78–1.42		0.749
Household income																
<$>\scale 80%\raster="rg1"<$> $60 000	1 (Reference)				1 (Reference)				1 (Reference)				1 (Reference)			
$28 000 to < $60 000	0.85	0.62–1.18		0.334	0.73	0.54–1.00		0.052	0.71	0.50–1.00		0.051	0.79	0.57–1.09		0.150
< $28 000	1.02	0.70–1.51		0.903	0.69	0.48–1.00		0.052	0.74	0.49–1.11		0.145	0.73	0.49–1.08		0.114
Employment status																
Regular	1 (Reference)				1 (Reference)				1 (Reference)				1 (Reference)			
Irregular/part-time	0.94	0.67–1.32		0.707	1.00	0.72–1.38		0.987	0.97	0.68–1.38		0.871	1.05	0.75–1.48		0.771
Self-employed	0.80	0.55–1.17		0.249	0.97	0.67–1.40		0.859	0.93	0.63–1.38		0.721	1.08	0.74–1.58		0.687
Unemployed	0.86	0.61–1.20		0.363	0.99	0.72–1.37		0.955	0.99	0.70–1.40		0.958	1.14	0.81–1.59		0.455

	Trust (newspapers)				Trust (magazines)				Trust (books)				Trust (healthcare privider)			
	OR		CI	p	OR		CI	p	OR		CI	p	OR		CI	p
Education																
College graduate	1 (Reference)				1 (Reference)				1 (Reference)				1 (Reference)			
Some college	0.91	0.64–1.30		0.613	1.26	0.90–1.78		0.177	1.20	0.83–1.72		0.328	0.99	0.54–1.84		0.985
High school or less	0.86	0.64–1.16		0.327	1.16	0.86–1.56		0.321	0.78	0.58–1.06		0.117	0.91	0.54–1.52		0.717
Household income																
<$>\scale 80%\raster="rg1"<$> $60 000	1 (Reference)				1 (Reference)				1 (Reference)				1 (Reference)			
$28 000 to < $60 000	0.83	0.60–1.15		0.251	0.74	0.54–1.01		0.060	0.79	0.56–1.10		0.157	0.40	0.19–0.83		0.013
< $28 000	0.58	0.39–0.85		0.005	0.67	0.46–0.97		0.032	0.66	0.45–0.98		0.037	0.29	0.13–0.63		0.002
Employment status																
Regular	1 (Reference)				1 (Reference)				1 (Reference)				1 (Reference)			
Irregular/part-time	0.91	0.65–1.28		0.599	0.84	0.61–1.17		0.299	0.81	0.57–1.13		0.213	1.36	0.75–2.47		0.317
Self-employed	0.76	0.52–1.11		0.158	0.83	0.57–1.21		0.333	0.69	0.47–1.01		0.055	0.79	0.44–1.41		0.417
Unemployed	0.93	0.67–1.31		0.691	0.86	0.62–1.19		0.367	0.95	0.67–1.33		0.748	0.99	0.57–1.73		0.981

	Trust (Internet website)				Trust (community newsletters)									
	OR		CI	p	OR		CI	p						
Education														
College graduate	1 (Reference)				1 (Reference)									
Some college	1.10	0.78–1.55		0.578	0.81	0.55–1.19		0.276						
High school or less	0.68	0.51–0.92		0.012	0.83	0.60–1.15		0.263						
Household income														
<$>\scale 80%\raster="rg1"<$> $60 000	1 (Reference)				1 (Reference)									
$28 000 to < $60 000	0.74	0.54–1.01		0.057	1.06	0.74–1.51		0.767						
<$28 000	0.73	0.50–1.07		0.109	0.89	0.59–1.34		0.567						
Employment status														
Regular	1 (Reference)				1 (Reference)									
Irregular/part-time	0.98	0.70–1.36		0.893	1.01	0.70–1.46		0.969						
Self-employed	0.65	0.44–0.95		0.026	0.65	0.44–0.97		0.034						
Unemployed	0.81	0.58–1.13		0.214	1.12	0.78–1.61		0.552						

Note: All models are additionally adjusted for gender, age, marital status, and subjective health status.

## Discussion

Inequality in health communication outcomes, defined as ‘how people access, seek, process, and act on heath information’, is one suggested link between SES and health inequality [6]. Thus, it is important to identify specific health communication outcomes that explain the influence of socioeconomic differences on health to decrease health disparities worldwide. To our knowledge, this is the first nationwide survey examining the relationship between social determinants of health and health communication outcomes in Japan.

This study identified health information seeking and self-efficacy to be patterned by education and income status. Our results were consistent with findings from the United States [7], providing support to the SIM’s proposed association between SES and different health communication outcomes [6]. This study identified people with unemployment to have a decreased likelihood of having recently sought health information, although linear association between employment status and health information seeking or self-efficacy was not found. The effect of employment status on health communication outcomes may have been masked in our study because a diverse range of participants such as students, stay-at-home parents, retired people, and job seekers were classified as either part-time workers or unemployed people. In Japan, the number of people who are not under regular employment or are unemployed has been increasing over the past decade, especially among the younger generation. This rapid change in the distribution of employment statuses will have an impact on health in the near future, indicating a need for further studies to examine the relationship between employment status and health communication outcomes as one of the potential mechanisms linking SES and health inequality.

A second implication of this study is the importance of considering health information sources when examining inequality in health communication outcomes. In the United States, it is reported that higher education status is related with lower trust in health information from mass media sources [7]. Our study results, however, indicate that exposure to, and trust in health information from mass media such as TV, radio, and newspapers are not patterned by SES. On the other hand, SES is identified to play a role in exposure to and trust in health information from interpersonal media such as healthcare providers and the Internet. Thus, as health communication exposure and use make the transition from traditional mass media to social media, health inequalities may widen. We also identified that trust in health information from community newsletters was higher compared with mass media or interpersonal media. In this Information Era when health information from various media are competing with each other, trust in information source is an important issue to consider. The effective use of community newsletters issued by the local government would therefore constitute part of influential communication strategies to address health of its community. As it stands, as only 18.2% of participants actually consumed health information from community newsletters, there is more potential to be tapped from this information source. Every municipality in Japan has its own public health center to promote health and prevent disease among its community. If each public health center is able to effectively deliver health information through community newsletters by using a social marketing approach, it might bring about a big effect on the health of the public.

A third important finding of this study is how exposure to and trust in health information are patterned by age and sex. Specifically, there was a statistically significant difference in sources of health information by sex and age. Female are more likely to report that TV information, friends and relatives, magazines, healthcare providers, and community newsletters are their sources of health information when compared with male. Also, the elderly are more likely to receive health information from newspapers, healthcare providers and community newsletters, whereas younger people receive it from TV news and Internet websites. This indicates a need for campaign and intervention planners to choose specific media channels depending on the intended target’s sex and age, rather than the mere use of mass media, in order to bring about consequential change in attitude and behavior.

This study has several limitations. First, cross-sectional data were used, and thus causality cannot be inferred. Future studies employing longitudinal design are required to examine the causal relationship among SES, health communication outcomes, and health status. Second, while not all confounders were accounted for, major confounders including sex, age, marital status and subjective health status were controlled statistically, thereby reducing chances of producing bias when examining the relationship between SES and health communication outcomes. The use of drop-off surveys to recruit study participants might have excluded those who spend little time at home, and it is conceivable that those who spend less time at home have different health communication outcomes, giving rise to a probable selection bias. However, further comparisons revealed the distribution of age, sex, regular employment, and subjective health to be equivalent between our selected sample and the general Japanese population. The distribution of education and household income belonging to that of the middle class were more frequent in our sample. Therefore, our analysis might have little alpha error and produce conservative results to test the association between health communication outcomes and socioeconomic status.

In summary, this study found that health communication outcomes are patterned by SES in Japan demonstrating the prevalence of health communication inequalities that could potentially link social determinants with health outcomes. Providing customized exposure to and enhancing the quality of health information while considering the social determinants of health may contribute to addressing social disparities in health in Japan.
